# Determination of cutoff values on computed tomography and magnetic resonance images for the diagnosis of atlantoaxial instability in small‐breed dogs

**DOI:** 10.1111/vsu.13799

**Published:** 2022-03-16

**Authors:** Bastien Planchamp, Franck Forterre, Beatriz Vidondo, Angel M. Hernandez‐Guerra, Ioannis N. Plessas, Martin J. Schmidt, Maja A. Waschk, Christina Precht

**Affiliations:** ^1^ Division of Small Animal Surgery, Department of Clinical Veterinary Medicine, Vetsuisse Faculty University of Bern Bern Switzerland; ^2^ Veterinary Public Health Institute, Department of Clinical Research and Veterinary Public Health, Vetsuisse Faculty University of Bern Liebefeld Switzerland; ^3^ Department of Veterinary Medicine and Surgery Universidad Cardenal Herrera‐CEU, CEU Universities Valencia Spain; ^4^ Davies Veterinary Specialists Limited Hitchin UK; ^5^ Clinic for Small Animals, Department of Veterinary Clinical Sciences, Justus‐Liebig‐ University Giessen Giessen Germany; ^6^ Clinic for Diagnostic Imaging, Vetsuisse Faculty University of Zürich Zürich Switzerland; ^7^ Clinical Radiology, Department of Clinical Veterinary Medicine, Vetsuisse Faculty University of Bern Bern Switzerland

## Abstract

**Objective:**

To determine cutoff values for the diagnosis of atlantoaxial instability (AAI) based on cross‐sectional imaging in small‐breed dogs.

**Study design:**

Retrospective multicenter study.

**Sample population:**

Client‐owned dogs (*n* = 123) and 28 cadavers.

**Methods:**

Dogs were assigned to three groups: a control group, a “potentially unstable” group, and an AAI‐affected group, according to imaging findings and clinical signs. The ventral compression index (VCI), cranial translation ratio (CTR), C1‐C2 overlap, C1‐C2 angle, atlantoaxial distance, basion‐dens interval, dens‐to‐axis length ratio (DALR), power ratio, and clivus canal angles were measured on CT or T2‐weighted magnetic resonance (MR) images. Receiver operating characteristic (ROC) analysis was performed to define cutoff values in flexed (≥25°) and extended (<25°) head positions.

**Results:**

Cutoff values for the VCI of ≥0.16 in extended and ≥0.2 in flexed head positions were diagnostic for AAI (sensitivity of 100% and 100%, specificity of 94.54% and 96.67%, respectively). Cutoff values for the other measurements were defined with a lower sensitivity (75%‐96%) and specificity (70%‐97%). A combination of the measurements did not increase the sensitivity and specificity compared with the VCI as single measurement.

**Conclusion:**

Cutoff values for several imaging measurements were established with good sensitivity and specificity. The VCI, defined as the ratio between the ventral and dorsal atlantodental interval, had the highest sensitivity and specificity in both head positions.

**Clinical significance:**

The use of defined cutoff values allows an objective diagnosis of AAI in small‐breed dogs. The decision for surgical intervention, however, should remain based on a combination of clinical and imaging findings.

## 1 INTRODUCTION

Atlantoaxial instability (AAI) is mostly observed in young small‐breed dogs and results in dorsal subluxation of the axis, leading to ventral compression of the cervical spine.^1^ It usually develops after minor trauma from congenital abnormalities of the dens axis and/or its supporting ligamentous structures.^1^, ^2^, ^3^, ^4^ Clinical signs in affected dogs range from cervical hyperesthesia to tetraplegia and, in severe cases, even respiratory arrest and death.^1^ Diagnosis of AAI is based on clinical findings combined with radiographs or cross‐sectional diagnostic imaging techniques such as computed tomography (CT) and magnetic resonance imaging (MRI).^1^, ^5^, ^6^ Radiographic findings include dorsal displacement of the dens axis within the vertebral canal, increased distance between the atlas dorsal arch and the axis spinous process, and increased angulation between both vertebrae, and hypoplasia or aplasia of the dens axis.^7^ Cummings et al. also recently published quantitative radiographic criteria for the diagnosis of AAI in small‐breed dogs.^7^ They concluded that a C1‐C2 overlap, defined as the distance of overlap between the axis spinous process and an atlas dorsal arch smaller than 1.55 mm was the most sensitive (100%) and specific (94.5%) radiographic measurement in the diagnosis of AAI in small‐breed dogs. Complementary pathological MRI findings include elongated and thickened apical, alar, or transverse ligaments, and focal spinal cord signal changes.^6^ In some cases the final diagnosis may nevertheless remain questionable even after the evaluation of the atlantoaxial joint on CT or MR images.

After evaluating the ligamentous functions and the influence of the head–neck position on imaging measurements used to assess the craniovertebral junction (CVJ) on CT and MR images, a combination of measurements was suggested to be promising for the diagnosis of AAI.^8^, ^9^ However, defined cutoff values for cross‐sectional imaging techniques, which allow an objective diagnosis of AAI in dogs, have not been established yet. Consequently, the goal of the present study was to determine cutoff values for the ventral compression index (VCI), cranial translation ratio (CTR), C1‐C2 overlap, C1‐C2 angle, atlantoaxial distance, basion‐dens interval, dens‐to‐axis length ratio (DALR), power ratio, and clivus canal angle, based on CT and MRI examinations of AAI‐affected and nonaffected small‐breed dogs. We hypothesized that a differentiation between AAI‐affected and nonaffected dogs would be clearly possible based on the imaging measurements mentioned above, consequently allowing an objective diagnosis of AAI in small‐breed dogs.

## 2 MATERIALS AND METHODS

### 2.1 Animals

Medical records of small‐breed dogs presented between January 2006 and August 2016 at 5 different institutions (Universities of Bern and Zürich, Switzerland, University Cardenal Herrera‐CEU Spain, Davies Veterinary Specialists United Kingdom, Justus‐Liebig‐University Giessen Germany) were retrospectively reviewed. The dogs affected by AAI dogs consisted of toy or small‐breed (<10 kg) dogs diagnosed with AAI on neurological examination and subjective interpretation of CT and/or MRI. Records were included if cross‐sectional imaging included the CVJ, encompassing at least the tuberculum sellae as a rostral anatomic landmark and the caudal endplate of C2 as caudal anatomic landmark. Controls consisted of small‐breed dogs presented in the institutions mentioned above during the same period of time for a disease not involving the CVJ. In addition, 28 small‐breed dog cadavers examined in a previous study in standardized head and neck positions were included.^9^ In control dogs, the CVJ was evaluated as unremarkable in CT and/or MRI studies. Dogs with an abnormal conformation of their atlantoaxial joint consisting in a subjective cranial translation of the dens axis in the absence of dorsal angulation were classified in a third group (a potentially unstable group). Dogs not meeting the inclusion criteria for any of the three groups (control group, “potentially unstable” group, and AAI‐affected group) were excluded. Cases and controls were not matched for breed, sex, or age. If the age or weight of an animal was not reported in the reviewed documentation, the parameter was indexed as a missing value. This study was performed in compliance with the Swiss ethics regulations and all owners provided a signed informed consent at the time of presentation or death in case of donation of their animals’ bodies to the furtherance of medical science.

### 2.2 Procedures and diagnostic imaging

Neurological and diagnostic imaging examinations were undertaken according to the particular institute's protocol and were therefore not standardized. However, cross‐sectional imaging had to include the CVJ as previously described. Images were sent in Digital Imaging and Communications in Medicine (DICOM) format and imported into the DICOM viewer IMPAXX EE (IMPAXX EE, Agfa Healthcare, Mortsel, Belgium) for review. Images were reviewed by the author (BP) under the supervision of a board‐certified veterinary radiologist (CP). No access to group information was available for the authors during the review and measurements.

### 2.3 Measurements

**FIGURE 1:**
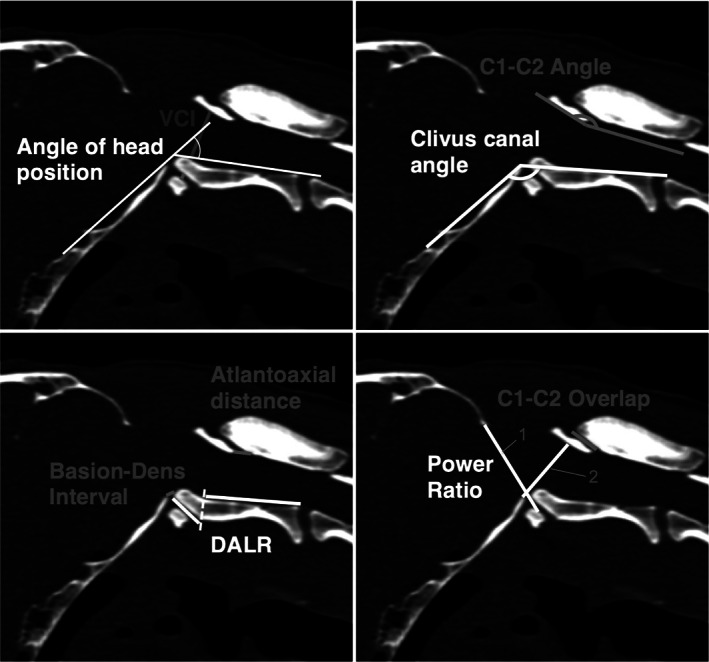
Overview of the quantitative imaging measurements. Opisthion‐C1 ventral arch interval (1) and basionQ15 C2 interval (2) to calculate the power ratio

**FIGURE 2:**
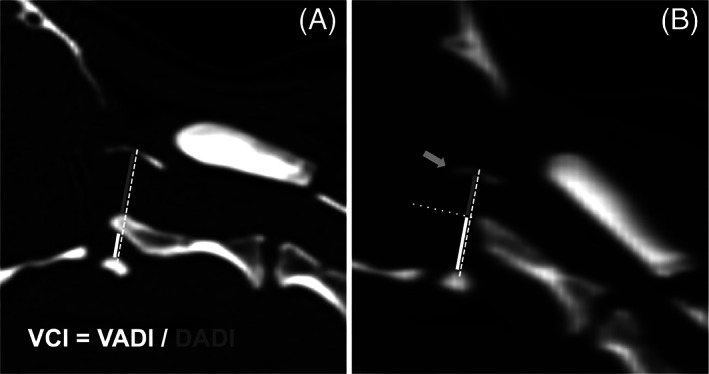
Method for the measurement of the ventral compression index (VCI). A line (white dashed) is drawn between the midpoints of the atlas ventral and dorsal arches. The ventral atlantodental interval (VADI, white) is measured along this line between the dorsal aspect of the atlas ventral arch and the ventral aspect of the dens axis. The dorsal atlantodental interval (DADI, gray) is measured along the same line between the dorsal aspect of the dens axis and the ventral aspect of the atlas dorsal arch (A). If the dens axis did not cross the line due to hypoplasia or dorsal angulation (B), a second line (white dotted) is drawn from the tip of the dens perpendicularly to the first line, defining the VADI and DADI. The VCI is calculated by dividing the VADI by the DADI. Note the severe occipital dysplasia in both cases and the atlantooccipital overlapping in case b (gray arrow)

**FIGURE 3:**
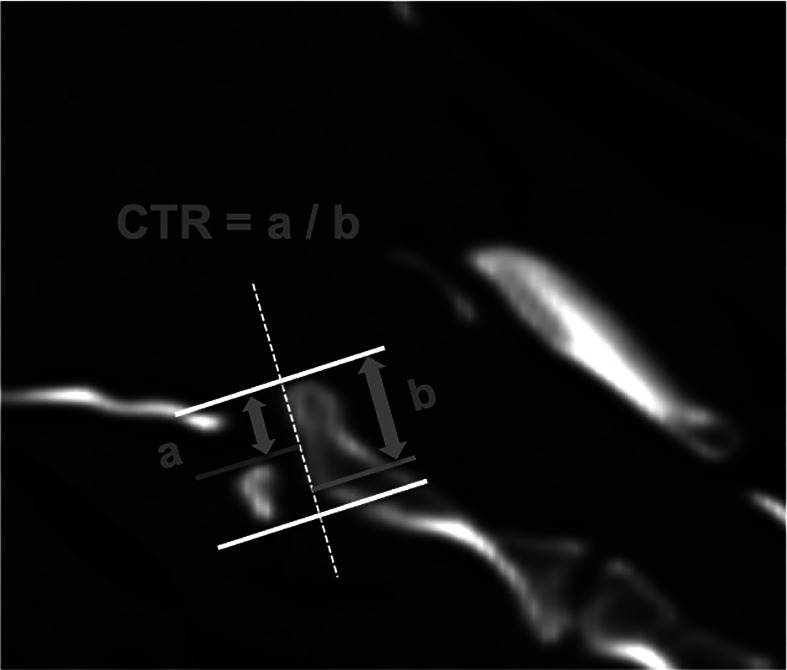
Method for the measurement of the cranial translation ratio (CTR). The distance of cranial translation (A) is measured parallel to the long axis of the dens axis (white dashed line), from the tip of the dens (white line) to the cranial border of the atlas ventral arch (gray line). The length of the dens axis (B) is defined as the distance between the tip (white line) and the ventral base of the dens (gray line). The CTR is then calculated by dividing the distance of cranial translation (A) by the length of the dens axis (B). A step formation occurs when the ventral base of the dens is located rostrally to the caudal border of the atlas ventral arch. In this situation, the gray line at the ventral base of the dens is not superimposed on the white line anymore

The measurements examined in the present study are derived from various radiological studies on AAI or Chiari‐like malformations in both human and veterinary medicine.^7^, ^10^, ^11^, ^12^, ^13^ The cranial translation ratio (CTR) was established specifically for the present study. Measurements are described in Table 1 and illustrated in Figures 1, 2, 3. The recorded value for each measurement was based on a single measurement. Cases and controls were classified into extended group or flexed group according to their head position during the diagnostic imaging. The head position was classified as extended when the angle of head position was <25° or as flexed when the angle was ≥25°. If both modalities, CT and MRI, were available, the measurements were performed on CT only. If MRI was the only modality available, the measurements were made on a T2‐weighted turbo spin echo (TSE) sagittal sequence. Qualitative parameters, such as medullary kinking, dorsal contact between the dens axis and spinal cord, cervical syringomyelia, and the transition of the base of the dens axis and the caudal border of the atlas ventral arch, were assessed during the evaluation of the diagnostic images to allow a better differentiation between groups.

**TABLE 1:** Description of the quantitative imaging measurements of the craniovertebral junction

Imaging measurements	Description
Angle of head position	Formed by the intersection of a line drawn between the tuberculum sellae and the basion and a second line between the cranio‐ and caudodorsal borders of the axis vertebral body
Clivus canal angle	Formed by the line extending from the top of the tuberculum sellae to the basion and the line parallel to the floor of the axis vertebral canal
C1‐C2 overlap	Cranial aspect of the axis spinous process to the caudal border of the atlas dorsal arch. A negative value indicates that the axis spinous process is no longer superimposed with the atlas arch.
Basion‐dens interval	Basion to the tip of the dens axis
Ventral compression index (VCI)	Calculated by dividing the ventral atlantodental interval (VADI) by the dorsal atlantodental interval (DADI), Figure 2.
Atlantoaxial distance	Caudal aspect of the atlas dorsal arch to the cranial aspect of the axis lamina
Power ratio	Calculated by dividing the distance between the basion and the midpoint of the atlas dorsal arch by the distance from the opisthion to the midpoint of the dorsal aspect of the atlas ventral arch
C1‐C2 angle	Formed by the intersection of a line parallel to the atlas dorsal arch and the axis lamina
Dens‐to‐axis length ratio (DALR)	Calculated by dividing the length of the dens by the one of the axis body. The length of the dens axis is defined as the distance between the tip and the ventral base of the dens. The length of the axis body is measured from the line drawn perpendicular to the axis body and passing through the base of the dens axis (white dashed line) to the caudal aspect of the axis vertebral body.
Cranial translation ratio (CTR)	Calculated by dividing the distance of cranial translation by the length of the dens axis (Figure 3).

Abbreviations: CTR, cranial translation ratio; DALR, dens‐to‐axis length ratio; DADI, dorsal atlantodental interval; VADI, ventral atlantodental interval; VCI, ventral compression index.

### 2.4 Statistical analysis

All statistical analyses were performed with NCSS 2021 statistical software (NCSS, LLC, Kaysville, Utah). Using PASS 2021 statistical software (PASS, LLC), we calculated the lower 1‐sided 95% confidence interval (CI) separately for both sample sizes – dogs examined in extension and in flexion. For the smaller group (dogs examined in flexion), a random sample of 23 subjects from the positive population (affected dogs) and 30 subjects from the negative population (controls) produced a 95% lower confidence bound with a distance of 0.078 from the sample area under the curve (AUC) of 0.800 to the lower limit, which was thus 0.722. For AUC = 0.99, the lower 1‐sided 95% CI was 0.99‐0.016 = 0.974. The lower 1‐sided 95% CI allows investigation of whether the AUC is greater than or equal to the estimate obtained. For all tests, *P*‐values below .05 (= desired α‐level) were considered to be significant. The assumption of a normal distribution of the measured variables was tested with Shapiro‐Wilk and D'Agostino Omnibus tests. First, descriptive statistics for each measurement were reviewed. Then, the statistical differences between the values obtained for the control group, the “potentially unstable” group, and the AAI group were assessed for every measured variable using a 2‐sample *t*‐test. Next, receiver operating characteristic (ROC) curve and cutoff analyses were performed on variables where there were significant differences between groups. Cutoff values were chosen to maximize sensitivity or to equilibrate sensitivity and specificity. After selection of the measured variables with the most relevant sensitivity and specificity, a multivariable logistic regression analysis was also carried out to evaluate if a combination of the variables could improve the accuracy of the diagnostic model. In addition, breed, sex, and weight differences were assessed using repeated measures analysis of variance with the dog's identification number as the subject variable.

## 3 RESULTS

A total of 151 small and toy breed dogs were included in the present study; 114 dogs examined at the University of Bern, 15 at the Davies Veterinary Specialists, 10 at the University of Zürich, 6 at the University Cardenal Herrera‐CEU, and 6 at the Justus‐Liebig‐University Giessen. The most common breeds were Yorkshire Terrier (*n* = 62) and Chihuahua (*n* = 57), followed by Papillon (*n* = 16), Maltese (*n* = 11), and various other breeds (*n* = 33) such as Pug, Pomeranian, Pinscher and Shih Tzu. Eighty‐seven dogs were male and 92 were female. Mean weight was 2.68 kg (SD ± 1.47 kg, missing values *n* = 35). No significant difference in weight was detected between groups with repeated measures of ANOVA. Mean age was 3.56 years (range from 3 months to 14 years old, missing values *n* = 28); dogs in the AAI group in flexed head position were younger with a mean of 1.78 year (range from 4 months to 7.5 years old, *P* = .042).

**FIGURE 4:**
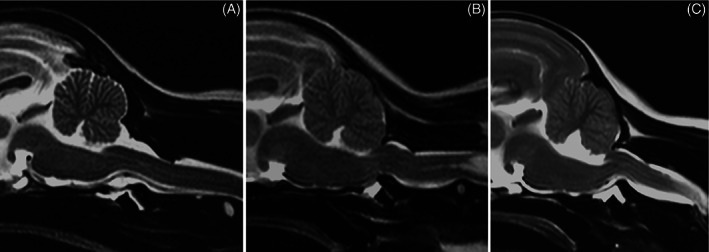
Sagittal MR images (T2‐weighted TSE) of the craniocervical junction of a Maltese with a physiologic atlantoaxial joint ((A) control, VCI 0.11, CTR 0), of a Yorkshire Terrier classified as potentially unstable ((B) VCI 0.13, CTR 0.35), and of a Chihuahua diagnosed with AAI ((C) VCI 0.3). Note the loss of continuity of the cerebrospinal fluid dorsal to the dens, focal spinal cord compression, and medullary kinking as well as the marked cervical syringomyelia (B)

Based on clinical and imaging findings, 55 dogs were classified as affected by AAI (AAI group) and 96 dogs were classified as unaffected by this condition (control group). Twenty‐one dogs were classified as potentially unstable (potentially unstable group) due to the abnormal conformation of their atlantoaxial joint with a subjective cranial translation of the dens axis in absence of dorsal angulation (Figure 4). Computed tomography was the only modality available for 47 dogs (33 in extended and 14 in flexed head position). Magnetic resonance imaging only was the only modality available for 62 dogs (51 extended and 11 flexed), and both modalities were available for 42 dogs (11 extended, 3 flexed, as well as for 28 cadavers from a previous study,^9^ which were evaluated in both positions). A total of 95 dogs and 28 cadavers were examined with the head in extension (angle of head position <25°, AAI group *n* = 32, control group *n* = 73, potentially unstable group *n* = 18), and 28 dogs and 28 cadavers in flexion (angle of head position ≥25°, AAI group *n* = 23, control group *n* = 30, potentially unstable group *n* = 3). The 3 dogs in the potentially unstable group examined in flexion were excluded from the statistical analysis due to the small number of cases in this group.

**FIGURE 5:**
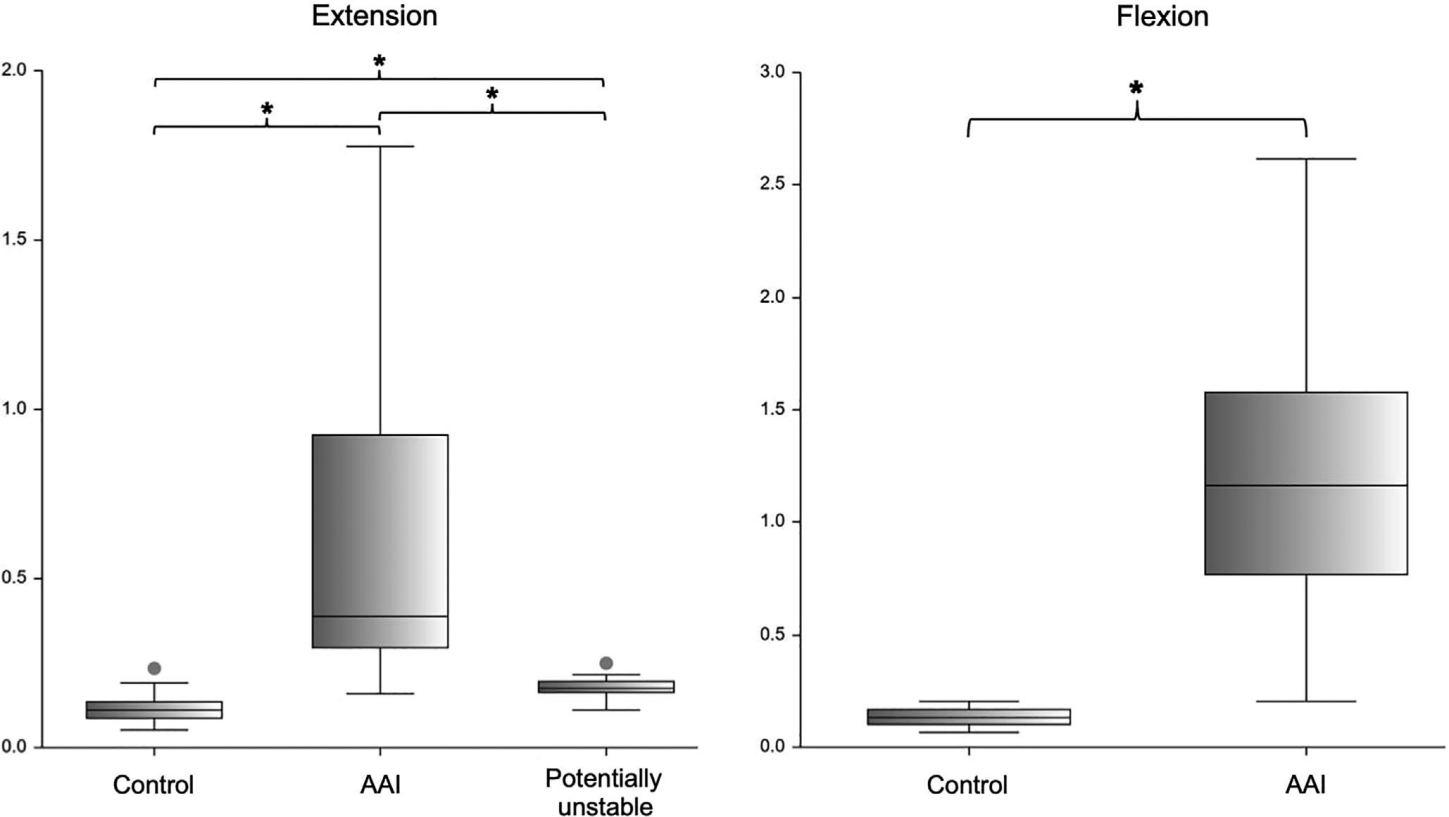
Box plot of the ventral compression index (VCI). The asterisk (*) over a brace indicates that both groups are significantly different. For every comparison done, *P* was .000001

**FIGURE 6:**
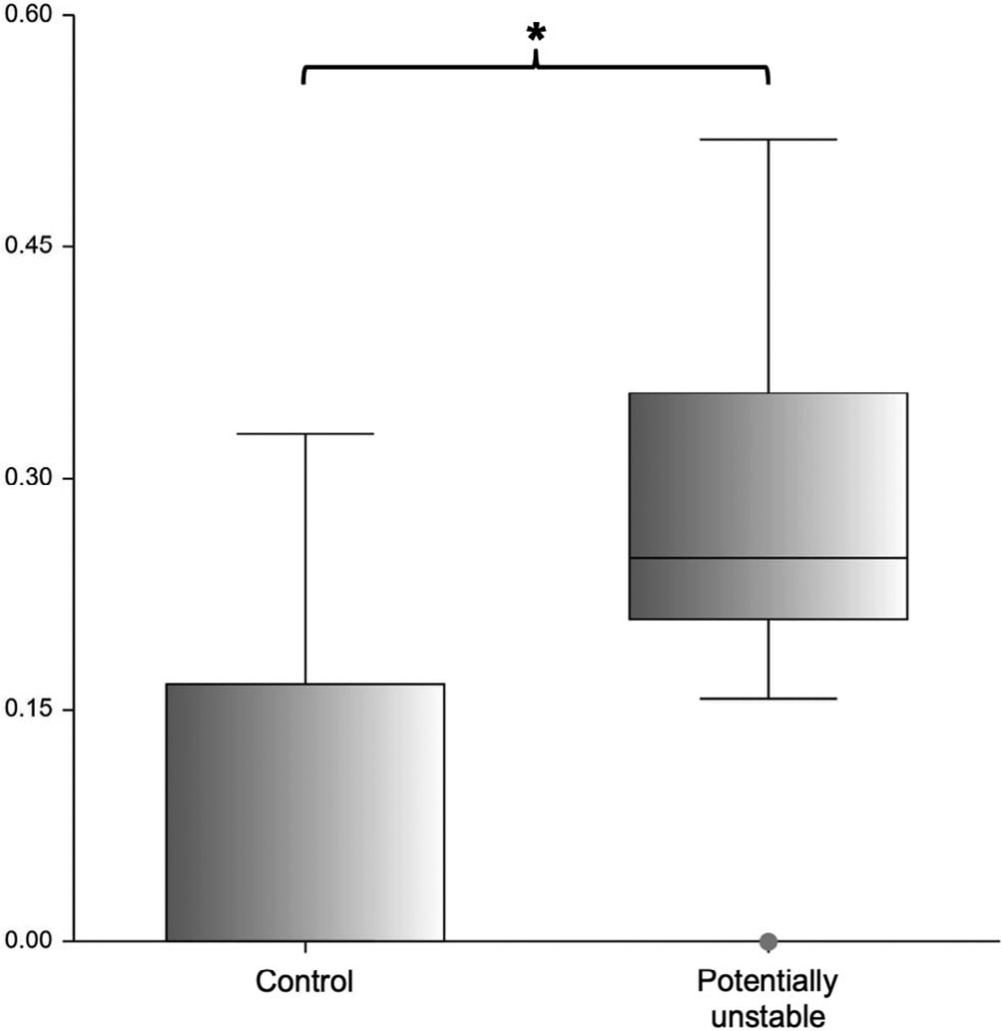
Box plot of the cranial translation ratio (CTR). The asterisk (*) over the brace indicates that both groups are different (*P* = .000001)

According to the results of the ROC analysis, high diagnostic power was identified for 6 of the measurements, namely the ventral compression index (VCI), C1‐C2 overlap, C1‐C2 angle, atlantoaxial distance, basion‐dens interval, and dens‐to‐axis length ratio (DALR) in both examined head positions (Table 2). The combination of these measurements in a multivariable logistic regression analysis did not increase the diagnostic power when compared with the results of VCI as a single measurement in both head positions. Mean ± standard deviation of the VCI in extended head position was 0.11 ± 0.03 for the control group, 0.62 ± 0.45 for the AAI group, and 0.18 ± 0.03 for the potentially unstable group. Mean ± standard deviation of the VCI in flexed head position was 0.13 ± 0.04 for the control group and 1.17 ± 0.61 for the AAI group (Figure 5). Differences between mean values of the three groups in extended or flexed head position were highly significant (for every comparison done, *P* was .000001). A VCI ≥0.16 in extension and ≥0.20 in flexion was diagnostic for AAI (sensitivity 100% and 100%, specificity 94.54% and 96.67%, AUC 0.9974 and 0.9986, respectively). The VCI did not appear to be dependent on breed, sex, or weight in our sample. The same measurements allowed differentiation between the AAI and the potentially unstable group (Table 3). However, it was impossible to differentiate the patients of the potentially unstable group from the ones in the control group with the measurements mentioned above. The cranial translation ratio (CTR) was the only measurement appropriate for this purpose. Mean ± standard deviation of the CTR was 0.07 ± 0.1 for the control group, and 0.28 ± 0.11 for the potentially unstable group (Figure 6). Forty‐one percent of the control dogs (*n* = 30) had a ratio of cranial translation of 0 versus only 1 dog in the potentially unstable group. A CTR ≥0.18 classified the patient as potentially unstable with a sensitivity of 90.48% and a specificity of 78.08% (AUC = 0.9054). Assessment of the atlantoaxial joint in potentially unstable patients using qualitative parameters also helps in the differentiation between these 2 groups. Medullary kinking was noted in every potentially unstable patient. Dorsal contact between the dens axis and the spinal cord evaluated according to the loss of continuity of the cerebrospinal fluid dorsal to the dens was present in 53% of the potentially unstable patient (missing data in 3 dogs, for which only CT was available) and in none of the control dogs that were assessed with MRI. A step formation between the base of the dens axis and the caudal border of the atlas ventral arch (Figure 3) and cervical syringomyelia were also often seen in potentially unstable patients (Figure 4).

**TABLE 2:** Results of the ROC analysis for the 6 measurements considered for the diagnosis of atlantoaxial instability (differentiation between control group and AAI group)

Measurements	Head Position	Cutoff value	Sensitivity (%)	Specificity (%)	PPV	AUC
**VCI**	Ext.	**≥0.16**	100	94.54	0.8889	0.9974
	Flex.	**≥0.20**	100	96.67	0.9583	0.9986
Atlantoaxial distance	Ext.	≥4.1	81.25	69.86	0.5417	0.8179
	Flex.	≥4.3	95.65	76.67	0.7586	0.9681
C1‐C2 Overlap	Ext.	≤2.7	84.38	80.82	0.6585	0.9110
	Flex.	≤1.8	95.65	90	0.88	0.9833
C1‐C2 Angle	Ext.	≥176.9	90.63	82.19	0.6905	0.9456
	Flex.	≥187.4	95.65	96.57	0.9565	0.9565
Basion‐Dens Interval	Ext.	≥5.9	75	73.97	0.5581	0.8399
	Flex.	≥3.0	95.65	96.67	0.9565	0.9957
DALR	Ext.	≤0.24	21.88	100	1.0	0.6607
	Flex.	≤0.29	21.74	100	1.0	0.7130

Abbreviations: AUC, area under the curve; DALR, dens‐to‐axis length ratio; PPV, positive predictive value; VCI, ventral compression index.

**TABLE 3:** Results of the ROC analysis for the differentiation between potentially unstable and AAI group

Measurements	Cutoff value	Sensitivity	Specificity	PPV	AUC
VCI	**≥0.23**	93.75	94.44	0.9677	0.9688
Atlantoaxial distance	≥4.5	65.63	66.67	0.7778	0.7248
C1‐C2 Overlap	≤1.8	68.75	66.67	0.7857	0.7743
C1‐C2 Angle	≥179.7	84.38	77.78	0.8710	0.8490
Basion‐Dens Interval	≥6.5	71.88	83.33	0.8846	0.7986
DALR	≤0.35	48.88	100	1.0	0.7309

Abbreviations: AUC, area under the curve; DALR, dens‐to‐axis length ratio; PPV, positive predictive value; VCI, ventral compression index.

Several measurements differed between control and AAI groups but were not suitable for diagnostic purposes. The clivus canal angle was found to be smaller in AAI affected dogs in extension (mean 167.2 vs. 174.1 in AAI nonaffected dogs, *P* = .006), but was not significantly different (*P* = .07) in flexion between both groups, limiting its use for the diagnosis of AAI. The power ratio was higher in AAI‐affected dogs (mean 0.9 vs. 0.82 in AAI‐nonaffected dogs in extension, *P* = .026, and 0.87 vs. 0.56 in AAI nonaffected dogs in flexion, *P* = .000001). However the sensitivity and specificity were lower than other parameters selected for the diagnosis of AAI (cutoff ≥0.85 in extension, sensitivity 62.5%, specificity 60.27%, AUC 0.6370 and cutoff ≥0.64 in flexion, sensitivity 91.3%, specificity 89.66%, AUC 0.9445). Furthermore, the opisthion‐C1 ventral arch interval mainly influences the ratio and is strongly dependent on the degree of occipital dysplasia. A detailed table with the descriptive statistics for all measurements is presented in the supplementary materials section.

## 4 DISCUSSION

We investigated the craniocervical junction of AAI‐affected and control dogs with the aim of determining diagnostic imaging cutoff values to promote an objective diagnosis of atlantoaxial instability in small‐breed dogs. Statistical analysis allowed the determination of cutoff values for 6 measurements used to diagnose AAI, namely the ventral compression index (VCI), C1‐C2 overlap, C1‐C2 angle, atlantoaxial distance, basion‐dens interval, and dens‐to‐axis length ratio (DALR). The most reliable measurement was the VCI defined as the ratio between the ventral and dorsal atlantodental interval, which quantify the degree of dorsal displacement of the dens axis in relation to the atlas. In human medicine, the atlantodental interval anterior to the dens axis, corresponding to the ventral atlantodental interval in dogs, is commonly used in the diagnosis of anterior atlantoaxial dislocations.^14^, ^15^ Due to the anatomical consistency in humans, only the anterior atlantodental interval could be measured for the diagnosis.^14^, ^16^ The ventral atlantodental interval (VADI) was shown to be significantly different between breeds and positively associated with the weight, the VCI is used in veterinary medicine as a relative measurement to overcome differences in patient size.^9^ We found that a VCI ≥0.16 in extension and ≥ 0.2 in flexion could be diagnostic for AAI in small‐breed dogs (sensitivity 100% and 100%, specificity 94.54% and 96.67%, respectively). Although it has not been investigated in the present study, intraobserver reproductibility and interobserver agreement have been reported to be good for the anterior and posterior atlantodental interval on plain radiographs and on CT in human medicine.^17^, ^18^ Furthermore, the VCI was neither dependent on the breed, sex, or weight, although, it was found to be larger in females than in males in a previous study^9^ (*P* = .035), but this point was not supported by the present study, where no difference was found (median 0.13 for both sexes). The previous result could be explained as most likely being due to a selection bias secondary to the small number of cases (*n* = 28).

Moreover, it was also shown that the VCI did not depend on the head position in control dogs as long as the head is not placed in maximal flexion.^9^ A VCI ≥0.2 in extension was associated with a sensitivity of 96.88% and a specificity of 98.63% and could also have been suggested as cutoff value. To use single cutoff value for both head positions is a simplification of the procedure, resulting in the possibility of using a single measurement and 1 cutoff value independently of the head position to determine AAI; however, this would be at the price of lower sensitivity. The decision to select 0.16 as cutoff value for the VCI in extended head position was taken to focus on the accurate identification of cases, because a missed diagnosis could lead to severe consequences for the patient. The repercussions for animals wrongly diagnosed positive due to a slightly lower specificity have a lesser impact because decision making for a surgical intervention should be based primarily on the severity of clinical signs and not only on diagnostic imaging findings. Cummings et al. already reported a higher VCI measured on a neutral lateral radiographic projection of the atlantoaxial joint in AAI affected toy breed dogs in comparison with control dogs (*P* = .0001).^7^ A cutoff value of ≥0.348 was 80% sensitive and 94.5% specific for AAI. The ventral and dorsal atlantodental interval were measured perpendicular to the longitudinal axis of the dens, leading to a total distance between the ventral and the dorsal arch of the atlas dependent on the dorsal angulation of the axis and consequently the degree of AAI. An adaptation of the measurement technique was proposed in a previous study.^9^ It is suggested that the total distance is measured between the midpoints of the ventral and dorsal atlas arches, respectively, and therefore is not influenced by the angulation of the axis in relation to the atlas. Applying this modification, the VCI is correlated to the position of the dens only. The difference in the measuring method of the VCI does not allow a direct comparison between our results and the results of Cummings et al. and could have contributed to the lower sensitivity obtained in that study.

The sensitivity and specificity for the cutoff values of the atlantoaxial distance, C1‐C2 overlap, C1‐C2 angle, and basion‐dens interval were lower than those for the VCI in both head positions. Although a combination of the measurements could not statistically increase the accuracy of the model for the diagnosis of AAI compared with the results from the VCI as a single measurement, determining the VCI might be sufficient to diagnose AAI in small‐breed dogs. If the diagnosis remains unclear, we recommend repeating the examination in a slightly flexed head position, which is considered to be closer to dogs' physiologic position in an awake state and could lead to dorsal angulation of the axis in cases with dynamic AAI.^19^, ^20^ A recent study by White et al. reports that a mild degree of cervical flexion could favor the diagnosis of AAI in dogs, which further supports this recommendation.^21^ A hyperflexion of the head is, however, contraindicated due to the risk of exacerbating focal pressure of the dens axis on the spinal cord in cases of AAI. Indeed, the ventral atlantodental interval as well as the VCI were shown to be higher in maximal flexion compared to slight flexion and extension in control dogs (*P* = .00001).^9^ In the same study, White et al. also fixed a cutoff value of >10° for the atlas to axis angle (AAA) when the head was placed in slight flexion,^21^ corresponding to >190° for the C1‐C2 angle in our study. The reported sensitivity and specificity were 90%, and 92% when this cutoff was evaluated in Yorkshire terriers, Chihuahuas or mixes of these breeds only. We defined a cutoff value of >187.4° for the C1‐C2 angle in flexion (sensitivity 95.65%, specificity 96.57%), which is a very similar finding.

A C1‐C2 overlap ≤2.7 mm in extension and ≤1.8 mm in flexion were found to be diagnostic for AAI (sensitivity 84.38% and 95.65%, specificity 80.82% and 90%, respectively). In a recent study, the C1‐C2 overlap was shown to be significantly different between breeds, but this difference was not related to the individual's weight difference,^9^ meaning that a cutoff value for each breed may be necessary to achieve a better sensitivity. However, a strong suspicion of AAI could be assumed, if the C1‐C2 overlap is negative. In the present study, a negative C1‐C2 overlap was identified in 37.5% of AAI‐affected dogs (12 of 32 dogs) in extension and in 82.6% (19 of 23 dogs) in flexion. In extension, only 1 negative C1‐C2 overlap of −2.9 mm was measured in a Yorkshire terrier in the control group (VCI 0.13), but none of the measurements were negative in control dogs when the head was placed in flexion. The same result was reported, with an even higher incidence, by Cummings et al., who found that a negative measurement predominated in 80% of the examined AAI‐affected dogs when the head was placed in extension. In the same study, a C1‐C2 overlap ≤1.55 mm was shown to be the most sensitive (100%) and specific (94.5%) radiographic measurement in the diagnosis of atlantoaxial instability on neutral lateral radiographs.^7^

Twenty‐one dogs were assigned to the potentially unstable group due to the abnormal conformation of their atlantoaxial joint, which consisted of a subjective cranial translation of the dens axis in the absence of dorsal angulation or subluxation. Medullary kinking, loss of continuity of the cerebrospinal fluid dorsal to the dens due to its contact with the cervical spinal cord, and syringomyelia were often associated imaging findings in the potentially unstable group. For this reason, we consider the cranial translation of the dens axis as a possible early stage of instability, although its clinical significance has not been clearly determined yet. Follow‐up studies of these patients and experimental studies aiming at surgically producing cranial translation of the dens by partial transsection of atlantoaxial ligaments may help to understand the significance of this observation. We hypothesize that the degree of cranial translation could be correlated with possible instability and propose a ratio to quantify the severity of the cranial translation. We found that a cranial translation ratio (CTR) ≥0.18 classified the patient as potentially unstable with a sensitivity of 90.48% and a specificity of 78.08%. The need to treat these patients depends on the severity of the clinical signs and on the published recommendations.^1^, ^2^ For the distinction between the potentially unstable and AAI groups the same measurements, that we found to be useful to differentiate between controls and AAI‐affected dogs, may be used applying different cutoff values. The VCI was again the most reliable measurement allowing differentiation between both groups. A VCI ≥0.23 was associated with a sensitivity of 93.75% and a specificity of 94.44%. The other measurements had a much lower sensitivity than for the differentiation between control and AAI‐affected dogs. This could be related to the higher variation in the position of the axis due to potential instability.

Dens axis hypoplasia or aplasia is a well described aspect of the pathophysiology of AAI in dogs.^1^ Takahashi et al. reported a lower dens‐to‐axis length ratio (DALR) in AAI‐affected dogs (mean 0.36) than in control dogs (mean 0.40, *P* = .001).^13^ This finding has been confirmed by the present study. The mean DALR was 0.34 for the AAI group and 0.39 for the control group in both head positions. The dens length was shorter in the AAI group than the control group (*P* = .00001), and the axis length was shorter in the AAI group than the control group (*P* = .00001). We found that patients with a markedly lower DALR are more susceptible to be affected by AAI. Furthermore, a DALR ≤0.24 was associated with a 100% specificity, but a sensitivity of only 21.88%. A DALR ≤0.35 was found to be 100% specific in the differentiation between the potentially unstable and AAI‐affected groups, but the specificity dropped when this cutoff value was used in the differentiation between AAI‐affected dogs and controls. For this reason, we recommend using the first cutoff value, DALR ≤0.24, as a diagnostic tool. In our sample, control dogs were often positioned with the head in hyperextension (negative angle of head position). As small‐breed dogs are commonly affected by occipital dysplasia, such a position is discouraged to avoid an atlantooccipital overlapping, which may lead to compression of the cerebellum by the atlas dorsal arch (Figure 2).^9^

The main limitations of the present study include the retrospective nature of the case selection and the lack of standardization of the imaging procedure and dog positioning. The distinction between the 2 head positions partially overcomes this. The cutoff values for the measurements examined have been determined based on a static CT and/or MRI study. In some cases, the use of the cutoff values mentioned above may not lead to a conclusive diagnosis and these dogs may need to be assessed by dynamic study. Further investigation is needed to establish an appropriate protocol for this purpose. The use of a mix of live dogs and cadavers for the control population may also be regarded as a limitation, although an influence on the range of motion of the CVJ appears unlikely as rigor mortis had already dissolved. The inclusion of cadavers could be justified by the difficulty in creating a size‐matched control population, especially for AAI‐affected dogs examined with the head in flexion. The power of the statistical analysis was also enhanced by the recruitment of cadavers (*n* = 28 in each head position).

In conclusion, the use of cutoff values for measurements in cross‐sectional imaging techniques allows an objective diagnosis of AAI in small‐breed dogs. A ventral compression index (VCI) ≥ 0.16 in extension and ≥0.2 in flexion was diagnostic for AAI (sensitivity 100% and 100%, specificity 94.54% and 96.67%, respectively). In the case of cranial translation of the dens axis without dorsal angulation or subluxation, subjective criteria like medullary kinking or cervical syringomyelia, as well as the use of the cranial translation ratio (CTR), help to quantify the degree of cranial translation as a possible early stage of atlantoaxial instability. A CTR ≥0.18 classifies the patient as potentially unstable with a sensitivity of 90.48% and a specificity of 78.08%. Decision making for surgical intervention however should remain based on the severity of clinical signs for both conditions and on published surgical recommendations.

## CONFLICT OF INTEREST

The authors declare no conflicts of interest related to this report.

## AUTHOR CONTRIBUTIONS

Bastien Planchamp: Contributed to conception of the study, study design, acquisition of the imaging data, measurements, and data analysis and interpretation. Drafted the manuscript. Christina Precht: Contributed to conception of the study, study design, acquisition of the imaging data, measurements, data analysis and interpretation. Franck Forterre: Contributed to the collection of the samples, conception of the study, and study design. Beatriz Vidondo: Contributed to the study design, data analysis, and interpretation. Maja A. Waschk: contributed to the acquisition of the imaging data, data analysis, and interpretation. Angel M. Hernandez‐Guerra: Contributed to the acquisition of the imaging data, data analysis, and interpretation. Ioannis N. Plessas: contributed to the acquisition of the imaging data, data analysis, and interpretation. Martin J. Schmidt: Contributed to the acquisition of the imaging data, data analysis and interpretation. All authors revised and approved the submitted manuscript.

## Supporting information

Supporting Information

## References

[1] Stalin C , Gutierrez‐Quintana R , Faller K , Guevar J , Yeamans C , Penderis J . A review of canine atlantoaxial joint subluxation. Vet Comp Orthop Traumatol. 2015;28(1):1‐8. doi:10.3415/VCOT-14-05-0064 25449605

[2] Lorinson D , Bright R , Thomas W , Selcer R , Wilkens B . Atlanto‐axial subluxation in dogs: the results of conservative and surgical therapy. Canine Pract. 1998;23(3):16‐18.

[3] Sanders SG , Bagley RS , Silver GM , Moore M , Tucker RL . Outcomes and complications associated with ventral screws, pins, and polymethyl methacrylate for atlantoaxial instability in 12 dogs. J Am Anim Hosp Assoc. 2004;40(3):204‐210. doi:10.5326/0400204 15131100

[4] Thomas WB , Sorjonen DC , Simpson ST . Surgical management of atlantoaxial subluxation in 23 dogs. Vet Surg. 1991;20(6):409‐412. 1369524 10.1111/j.1532-950x.1991.tb00348.x

[5] Parry AT , Upjohn MM , Schlegl K , Kneissl S , Lamb CR . Computed tomography variations in morphology of the canine atlas in dogs with and without atlantoaxial subluxation. Vet Radiol Ultrasound. 2010;51(6):596‐600. 21158229 10.1111/j.1740-8261.2010.01711.x

[6] Middleton G , Hillmann DJ , Trichel J , Bragulla HH , Gaschen L . Magnetic resonance imaging of the ligamentous structures of the occipitoatlantoaxial region in the dog. Vet Radiol Ultrasound. 2012;53(5):545‐551. doi:10.1111/j.1740-8261.2012.01960.x 22730919

[7] Cummings KR , Grosso F , Moore GE , Rochat MA , Thomovsky S , Bentley R . Objective measurements of the atlantoaxial joint on radiographs performed without flexion can increase the confidence of diagnosis of atlantoaxial instability in toy breed dogs. 2018;59. 10.1111/vru.12668 30014570

[8] Planchamp B , Bluteau J , Stoffel MH , Precht C , Schmidli F , Forterre F . Morphometric and functional study of the canine atlantoaxial joint. Res Vet Sci. 2020;128:76‐85. doi:10.1016/j.rvsc.2019.11.005 31759272

[9] Planchamp B , Forterre F , Vidondo B , Beugger A , Müller A , Precht C . Influence of the head neck position on imaging measurements used to assess the Craniovertebral junction in small breed dogs: a cadaveric study. Vet Comp Orthop Traumatol. 2021;34(4):268‐278. doi:10.1055/s-0041-1726081 33979877

[10] Waschk MA , Vidondo B , Carrera I , et al. Craniovertebral junction anomalies in small breed dogs with Atlantoaxial instability: a multicentre case‐control study. Vet Comp Orthop Traumatol. 2019;32(1):33‐40. 30485878 10.1055/s-0038-1675797

[11] Smoker WR . Craniovertebral junction: normal anatomy, craniometry, and congenital anomalies. Radiogr Rev Publ Radiol Soc N Am Inc. 1994;14(2):255‐277. doi:10.1148/radiographics.14.2.8190952 8190952

[12] Rojas CA , Bertozzi JC , Martinez CR , Whitlow J . Reassessment of the Craniocervical junction: Normal values on CT. Am J Neuroradiol. 2007;28(9):1819. doi:10.3174/ajnr.A0660 17893223 PMC8134200

[13] Takahashi F , Hakozaki T , Kanno N , Harada Y , Yamaguchi S , Hara Y . Evaluation of the dens‐to‐axis length ratio and dens angle in toy‐breed dogs with and without atlantoaxial instability and in healthy beagles. Am J Vet Res. 2017;78(12):1400‐1405. doi:10.2460/ajvr.78.12.1400 29182395

[14] Yang SY , Boniello AJ , Poorman CE , Chang AL , Wang S , Passias PG . A review of the diagnosis and treatment of atlantoaxial dislocations. Glob Spine J. 2014;4(3):197‐210. doi:10.1055/s-0034-1376371 PMC4111952 25083363

[15] Bono CM , Vaccaro AR , Fehlings M , et al. Measurement techniques for upper cervical spine injuries: consensus statement of the spine trauma study group. Spine. 2007;32(5):593‐600. doi:10.1097/01.brs.0000257345.21075.a7 17334296

[16] Greenberg AD . Atlanto‐axial dislocations. Brain J Neurol. 1968;91(4):655‐684. doi:10.1093/brain/91.4.655 5704829

[17] Wellborn CC , Sturm PF , Hatch RS , Bomze SR , Jablonski K . Intraobserver reproducibility and interobserver reliability of cervical spine measurements. J Pediatr Orthop. 2000;20(1):66‐70. 10641692

[18] Yoon K , Cha S , Ryu JA , Park D , Lee S , Joo K . Anterior Atlantodental and posterior Atlantodental intervals on plain radiography, multidetector CT, and MRI. J Korean Soc Radiol. 2015;72:57. doi:10.3348/jksr.2015.72.1.57

[19] Cerda‐Gonzalez S , Dewey CW , Scrivani PV , Kline KL . Imaging features of atlanto‐occipital overlapping in dogs. Vet Radiol Ultrasound. 2009;50(3):264‐268. 19507388 10.1111/j.1740-8261.2009.01531.x

[20] Upchurch JJ , McGonnell IM , Driver CJ , Butler L , Volk HA . Influence of head positioning on the assessment of Chiari‐like malformation in cavalier king Charles spaniels. Vet Rec. 2011;169(11):277. doi:10.1136/vr.d4395 21824898

[21] White D , Renberg W , Roush J , Hallman M , Mauler D , Milliken G . Flexed radiographic angles for determination of atlantoaxial instability in dogs. Vet Surg. 2019;48(8):1406‐1415. doi:10.1111/vsu.13323 31506972

