# Supplementary material for: Determination of cutoff values on computed tomography and magnetic resonance images for the diagnosis of atlantoaxial instability in small‐breed dogs
Source: Vet Surg. 2022 Mar 16;51(4):620–30. doi: 10.1111/vsu.13799 (PMC9311214; doi:10.1111/vsu.13799)
Supplement: Supplementary file 1 — Supporting Information [file VSU-51-620-s001.docx]

| **Measurements** | **Groups** | **Mean** | **Standard Deviation** | **Minimum** | **Maximum** | **Range** |
| --- | --- | --- | --- | --- | --- | --- |
| Angle of head position | 0* | 1.65 | 13.02 | -25.8 | 23.69 | 49.49 |
|  | 1* | 7.87 | 10.3 | -15.4 | 24 | 39.4 |
|  | 2 | 7.11 | 9.59 | -15.6 | 22.2 | 37.8 |
| Clivus canal angle | 0* | 174.14 | 12.24 | 151.02 | 200.6 | 49.58 |
|  | 1* | 167.16 | 10.02 | 154.5 | 190.9 | 36.4 |
|  | 2 | 169.9 | 9.27 | 155.7 | 191.1 | 35.4 |
| VCI | 0*** | 0.115 | 0.033 | 0.051 | 0.235 | 0.184 |
|  | 1*** | 0.619 | 0.45 | 0.16 | 1.775 | 1.615 |
|  | 2*** | 0.176 | 0.03 | 0.113 | 0.25 | 0.137 |
| VADI | 0*** | 0.97 | 0.29 | 0.44 | 1.9 | 1.46 |
|  | 1*** | 3.41 | 1.77 | 1.42 | 7.1 | 5.68 |
|  | 2*** | 1.3 | 0.27 | 0.87 | 1.9 | 1.03 |
| DADI | 0*** | 8.45 | 1.05 | 6.4 | 11.4 | 5.0 |
|  | 1*** | 6.375 | 1.78 | 3.4 | 11.9 | 8.5 |
|  | 2*** | 7.37 | 0.83 | 6.3 | 9.7 | 3.4 |
| Atlantoaxial Distance | 0*** | 3.51 | 1.58 | 1.1 | 8.6 | 7.5 |
|  | 1*** | 5.44 | 1.55 | 2.9 | 9.2 | 6.3 |
|  | 2*** | 4.14 | 1.25 | 2.2 | 6.4 | 4.2 |
| C1-C2 Overlap | 0*** | 4.4 | 2.15 | -2.9 | 9.4 | 12.3 |
|  | 1*** | 0.62 | 2.16 | -4.3 | 4.1 | 8.4 |
|  | 2*** | 2.71 | 1.65 | -0.38 | 6.7 | 7.08 |
| C1-C2 Angle | 0*** | 169.34 | 7.89 | 151.5 | 184.4 | 32.9 |
|  | 1*** | 190.82 | 11.6 | 172.6 | 215.6 | 43 |
|  | 2*** | 176.46 | 8.34 | 156.1 | 190.7 | 34.6 |
| Basion-Dens Interval | 0*** | 4.97 | 2.14 | 1.18 | 13.3 | 12.12 |
|  | 1*** | 7.72 | 1.98 | 3.7 | 11.3 | 7.6 |
|  | 2*** | 5.58 | 1.1 | 3.4 | 7.6 | 4.2 |
| DALR | 0* | 0.389 | 0.033 | 0.29 | 0.477 | 0.187 |
|  | 1*, ** | 0.343 | 0.092 | 0.169 | 0.51 | 0.34 |
|  | 2 ** | 0.4 | 0.019 | 0.355 | 0.44 | 0.086 |
| Dens Length | 0* | 6.7 | 1.2 | 4.5 | 10.8 | 6.3 |
|  | 1*, ** | 4.93 | 1.59 | 2.0 | 8.3 | 6.3 |
|  | 2 ** | 6.17 | 0.73 | 5.0 | 8.3 | 3.3 |
| Axis Length | 0*, ** | 17.3 | 3.3 | 11.5 | 29.2 | 17.7 |
|  | 1* | 14.38 | 2.89 | 7.9 | 21.9 | 14 |
|  | 2 ** | 15.5 | 2.49 | 12.5 | 23.4 | 10.9 |
| Power ratio | 0*, ** | 0.816 | 0.192 | 0.42 | 1.22 | 0.794 |
|  | 1* | 0.896 | 0.157 | 0.62 | 1.24 | 0.62 |
|  | 2 ** | 0.955 | 0.224 | 0.54 | 1.32 | 0.78 |
| Basion-C2 Interval | 0 | 13.7 | 2.11 | 9.92 | 22.7 | 12.78 |
|  | 1 | 13.61 | 1.54 | 11.1 | 18.3 | 7.2 |
|  | 2 | 13.64 | 1.56 | 11.5 | 18 | 6.5 |
| Opisthion-C1 ventral arch Interval | 0*, ** | 17.37 | 3.17 | 11.7 | 26.04 | 14.34 |
|  | 1* | 15.56 | 2.81 | 10.7 | 23 | 12.3 |
|  | 2 ** | 15.0 | 3.64 | 9.7 | 21.6 | 11.9 |

**Supplementary Material**

**Table 1**: Mean, standard deviation, minimum, maximum and range of the measurements when the head was placed in extension (angle of head position < 25°)

Total of dogs n = 123: control group (0) n = 73, AAI group (1) n = 32, potentially instable group (2) n = 18.
The asterisks (*, **) indicate when two groups are significantly different. Three asterisks (***) indicate that all groups are significantly different among each other.

| **Measurements** | **Groups** | **Mean** | **Standard Deviation** | **Minimum** | **Maximum** | **Range** |
| --- | --- | --- | --- | --- | --- | --- |
| Angle of head position | 0* | 48.63 | 1.45 | 30.9 | 62.92 | 32.02 |
|  | 1* | 41.25 | 13.93 | 26.9 | 76.8 | 49.9 |
| Clivus canal angle | 0 | 128.85 | 7.81 | 115.58 | 146.9 | 31.32 |
|  | 1 | 135.07 | 14.16 | 108.7 | 154.5 | 45.8 |
| VCI | 0* | 0.135 | 0.039 | 0.063 | 0.206 | 0.143 |
|  | 1* | 1.174 | 0.607 | 0.203 | 2.618 | 2.414 |
| VADI | 0* | 1.089 | 0.302 | 0.55 | 1.59 | 1.04 |
|  | 1* | 5.087 | 2.46 | 1.2 | 10.7 | 9.5 |
| DADI | 0* | 8.16 | 0.86 | 6.6 | 10.37 | 3.77 |
|  | 1* | 4.71 | 1.5 | 1.9 | 7.5 | 5.6 |
| Atlantoaxial distance | 0* | 3.51 | 1.07 | 1.65 | 5.65 | 4.0 |
|  | 1* | 7.56 | 2.03 | 3.7 | 12.9 | 9.2 |
| C1-C2 Overlap | 0* | 4.01 | 1.37 | 1.5 | 6.2 | 4.7 |
|  | 1* | -2.82 | 2.56 | -6.3 | 3.2 | 9.5 |
| C1-C2 Angle | 0* | 171.63 | 7.9 | 154.21 | 189.53 | 35.32 |
|  | 1* | 209.4 | 17.87 | 156.8 | 244.2 | 87.4 |
| Basion-Dens Interval | 0* | 1.63 | 0.7 | 0.35 | 3.06 | 2.71 |
|  | 1* | 8.14 | 3.49 | 2.9 | 19.5 | 16.6 |
| DALR | 0* | 0.388 | 0.03 | 0.29 | 0.46 | 0.17 |
|  | 1* | 0.342 | 0.07 | 0.194 | 0.45 | 0.25 |
| Dens length | 0* | 6.845 | 0.97 | 5.26 | 8.6 | 3.34 |
|  | 1* | 4.48 | 1.05 | 2.6 | 6.0 | 3.4 |
| Axis length | 0* | 17.65 | 2.3 | 14.2 | 22.68 | 8.48 |
|  | 1* | 13.43 | 3.44 | 7.4 | 19.6 | 12.2 |
| Power ratio | 0* | 0.56 | 0.07 | 0.385 | 0.692 | 0.307 |
|  | 1* | 0.865 | 0.17 | 0.479 | 1.162 | 0.683 |
| Basion-C2 Interval | 0* | 11.09 | 0.86 | 9.39 | 13.55 | 4.16 |
|  | 1* | 12.27 | 2.46 | 8.1 | 18.7 | 10.6 |
| Opisthion-C1 ventral arch Interval | 0* | 20.02 | 2.45 | 15.55 | 27.05 | 11.5 |
|  | 1* | 14.39 | 2.23 | 10.1 | 19.6 | 9.5 |

**Table 2**: Mean, standard deviation, minimum, maximum and range of the measurements when the head was placed in flexion (angle of head position ≥ 25°)

Total of dogs n = 56: control group (0) n = 30, AAI group (1) n = 23, potentially instable group (excluded from the statistical analysis) n = 3

The asterisk (*) indicate when the two groups are significantly different.
